# Medicinal Cannabis: *In Vitro* Validation of Vaporizers for the Smoke-Free Inhalation of Cannabis

**DOI:** 10.1371/journal.pone.0147286

**Published:** 2016-01-19

**Authors:** Christian Lanz, Johan Mattsson, Umut Soydaner, Rudolf Brenneisen

**Affiliations:** 1 Department of Clinical Research, Laboratory of Phytopharmacology, Bioanalytics and Pharmacokinetics, University of Bern, Bern, Switzerland; 2 Center of Laboratory Medicine, University Institute of Clinical Chemistry, Inselspital, University Hospital Bern, Bern, Switzerland; Martin Luther University, GERMANY

## Abstract

Inhalation by vaporization is a promising application mode for cannabis in medicine. An *in vitro* validation of 5 commercial vaporizers was performed with THC-type and CBD-type cannabis. Gas chromatography/mass spectrometry was used to determine recoveries of total THC (THC_tot_) and total CBD (CBD_tot_) in the vapor. High-performance liquid chromatography with photodiode array detection was used for the quantitation of acidic cannabinoids in the residue and to calculate decarboxylation efficiencies. Recoveries of THC_tot_ and CBD_tot_ in the vapor of 4 electrically-driven vaporizers were 58.4 and 51.4%, 66.8 and 56.1%, 82.7 and 70.0% and 54.6 and 56.7% for Volcano Medic^®^, Plenty Vaporizer^®^, Arizer Solo^®^ and DaVinci Vaporizer^®^, respectively. Decarboxylation efficiency was excellent for THC (≥ 97.3%) and CBD (≥ 94.6%). The gas-powered Vape-or-Smoke^™^ showed recoveries of THC_tot_ and CBD_tot_ in the vapor of 55.9 and 45.9%, respectively, and a decarboxylation efficiency of ≥ 87.7 for both cannabinoids. However, combustion of cannabis was observed with this device. Temperature-controlled, electrically-driven vaporizers efficiently decarboxylate inactive acidic cannabinoids and reliably release their corresponding neutral, active cannabinoids. Thus, they offer a promising application mode for the safe and efficient administration of medicinal cannabis.

## Introduction

Cannabis has an unrivaled history of continuous cultivation, which started in old China in Neolithic times 6000 years ago. It was used as important fiber plant and also as medicine in these times [1]. A thorough scientific evaluation of the medical use of cannabis goes back to the work of Sir William B. O’Shaughnessy in 1838–1840 [2]. Since then cannabis has been intensively investigated. In the early 1960s cannabidiol (CBD) and the most psychoactive cannabinoid delta-9-tetrahydrocannabinol (THC) were identified [3,4]. Until 2009, more than 525 constituents were discovered, among them about 100 different cannabinoids [5–7]. Milestones were in the late 1980s and early 1990s the identification and cloning of the cannabinoid 1 receptor (CB1R) in brain tissue and the discovery of a peripheral cannabinoid 2 receptor (CB2R) [8–10]. Growing evidence has been obtained in recent years that the cannabinoid receptor system plays a central role in the regulation of many key functions to maintain homeostasis [11]. Many attempts have been made to mitigate and treat symptoms of a large variety of diseases *via* the activation or inhibition of the cannabinoid receptors [8]. THC (dronabinol), which is a partial agonist to CB1R and to a smaller extent also to CB2R, is available in many countries for several indications [12]. It is administered orally to treat pain, nausea, spasticity, and loss of appetite. It has proven to be effective in patients suffering from cancer, multiple sclerosis, amyotrophic lateral sclerosis, chronic pain, and other diseases [7,12,13]. However, THC may cause dose-dependent psychotropic side-effects [12]. In parallel, an increasing interest in the medical use of cannabis was observed, too [13]. Besides THC, other cannabinoids and also non-cannabinoids, such as terpenoids, most probably contribute to and modulate the overall pharmacological effects of cannabis [13–16]. Numerous recent studies have proven the anti-inflammatory and neuroprotective properties of THC and CBD, another major phytocannabinoid [17]. Furthermore, CBD is known to reduce the psychotropic effects of THC [18]. In addition, THC and CBD act synergistically [18,19]. An important aspect of the use of cannabinoids in medicine and the evaluation of administration modes are the particular pharmacokinetic properties of these highly lipophilic compounds. Compared to the very rapid and good absorption after pulmonal administration, the absorption after oral administration is slow, unpredictable, and erratic. A percentage of 6–20% shows the very low systemic oral bioavailability of THC and other cannabinoids, which is due to the sensitivity of THC to the acidic gastric fluid and an extensive first pass metabolism in gut and liver. Furthermore, the bioavailability after oral administration was reported to be inter-individually very variable, thus showing an unreliable onset of action [13,20,21]. Blood concentrations of THC were reported to be 25–30% of those resulting after smoking the same dose [21].

A further aspect has to be taken into account concerning the use of medicinal cannabis. THC and CBD are present in the plant as THC acid A (THCA-A) and CBD acid (CBDA), respectively, which are pharmacologically inactive and have therefore to be converted first to the active neutral compounds. The decarboxylation is temperature-dependent and occurs at high temperatures > 180°C, which is the reason why cannabis is traditionally smoked for recreational purposes [22]. However, smoking of cannabis is potentially harmful and thus not acceptable for therapeutic purposes. Therefore, and considering the bad oral pharmacokinetics of cannabinoids, alternative administration techniques are needed [13,15,21], such as inhalation by vaporizing cannabis or cannabinoids. This efficient and less harmful pulmonal application mode is also interesting for patients who are not able to swallow. For patients suffering from heavy respiratory tract diseases, the use of vaporizers should be carefully evaluated. In such cases an alternative option could be oromucosal cannabis or cannabinoid preparations (sublingual sprays and tablets).

Vaporizers decarboxylate cannabinoid acids at about 200°C and release neutral, volatile cannabinoids, which enter the systemic circulation *via* pulmonary absorption from the vapor [22]. The non-pyrolytic vaporization avoids the formation of hazardous combustion products, such as tar, polycyclic aromatic hydrocarbons (PAH), carbon monoxide, and other carcinogens (e.g. benzene) [22–25]. Gieringer and co-workers demonstrated the advantages of cannabis vaporization compared to smoking and showed that the formation of combustion products is suppressed almost completely. However, the release of cannabinoids into the vapor is dependent on the device used [23–25]. Thirty-six to 61% of THC present in cannabis was found in the vapor using the Volcano Medic^®^ vaporizer at 226°C. Only 3 non-cannabinoids were found in the vapor. In contrast, about 150 chemicals were identified in the smoke of combusted cannabis, among them 5 PAHs, known as strong carcinogens [25]. Fischedick and co-workers performed a comprehensive comparison between cannabis smoke and cannabis vapor using Volcano Medic^®^ [26]. They demonstrated the absence of pyrolysis products in the vapor at 200°C and that the efficiency of volatilization is higher with the vaporizer than the cigarette. Several groups performed a comprehensive evaluation of Volcano Medic^®^. Hazekamp and co-workers defined the optimal parameters for the vaporization of cannabis [22]. The temperature-dependent delivery of THC into the vapor was demonstrated by Pomahacova and colleagues, who found the highest yield of THC and the optimum ratio of cannabinoids-to-harmful by-products at 230°C [27]. A pharmacokinetic and pharmacodynamic evaluation of the same vaporizer was performed by Abrams and co-workers [28]. They showed comparable pharmacokinetic and pharmacodynamic results between smoking and vaporizing cannabis, whereas the production of carbon monoxide, as an indicator of combustion, was completely suppressed using the vaporizer. The efficacy of pulmonary absorption of inhaled THC has also been demonstrated by Naef and colleagues, comparing intravenously administered THC to the pulmonary absorption of THC released as liquid aerosol from a pressure-driven nebulizer [29]. Further pharmacodynamic data supporting the efficacy of pulmonary absorption are available from pure THC vaporized with Volcano Medic^®^ [30]. In addition, evidence for the clinical usefulness, acceptance and advantages of vaporizing THC instead of smoking was shown by 2 studies investigating pulmonary symptoms. One concluded from a questionnaire remarkably decreased respiratory problems like cough, phlegm and tightness in the chest in cannabis users who vaporized cannabis [31]. The other study monitored pulmonary function with spirometry among cannabis smokers prior to and following the use of a cannabis vaporizer [32]. It showed an improvement and normalization of the pulmonary function within one month.

To the best of our knowledge Volcano Medic^®^ is nowadays the only vaporizer, which has been thoroughly validated for the vaporization of cannabis according to scientific standards and no comparison with other vaporizers has been published so far. Therefore, the aim of the present study was to investigate *in vitro* the performance of 3 pocket-size and 1 hand-held vaporizers commercially available on the Swiss market in comparison with the benchtop vaporizer Volcano Medic^®^ as the gold standard.

## Materials and Methods

### Chemicals

The following calibrated deuterated and non-deuterated standards were obtained from Lipomed (Arlesheim, Switzerland): THC (1 mg/mL in ethanol), cannabinol (CBN; 1 mg/mL in methanol), THCA-A (1 mg/mL in isopropanol), THC-D3 (0.1 mg/mL in ethanol) and CBN-D3 (0.1 mg/mL in methanol). CBD and CBD-D3 were purchased from THC Pharm (Frankfurt a.M., Germany) as powder and calibrated standard (1 mg/mL in methanol), respectively. Cannabichromene (CBC) was from Toronto Research Chemicals (Toronto, Ontario, Canada). CBDA was supplied by ReseaChem (Burgdorf, Switzerland) and synthetic THC (dronabinol) was obtained from Hänseler (Herisau, Switzerland). All standards were diluted in MeOH to obtain calibrator and control samples of the desired concentrations. Female flower tops of THC-type cannabis were provided from Bedrocan BV (Veendam, The Netherlands), CBD-type cannabis was supplied from a local grower. All chemicals and solvents were of analytical and HPLC grade, respectively, and obtained either from Sigma-Aldrich Chemie (Buchs, Switzerland) or Merck (Darmstadt, Germany). Polypropylene cartridges (Chromabond, 15 mL) for solid phase extraction (SPE) were supplied by Macherey-Nagel (Oensingen, Switzerland) and LiChroprep RP-18 (40–63 μm) used as the sorbent was from Merck.

### Preparation and quantitation of test materials (cannabis, cannabinoid standards)

Dried cannabis was grinded and homogenized carefully in a mortar and stored at 4°C. Five 100-mg aliquots of each cannabis type were weighed into 10-mL glass vials with Teflon-coated screw caps (Infochroma, Zug, Switzerland) and combined with 1.0 mL of methanol containing 10% (v/v) of chloroform (MeOH-CHCl_3_ 9:1). The vials were tightly closed and the samples extracted for 15 min in an ultrasonic bath at room temperature. After filtration by using a Pasteur pipette with glass wool the extract was diluted 10 times with MeOH. Ten μL of this dilution were combined with 25 μL of the internal standard solution (IS; THC-D3, CBD-D3 and CBN-D3; 40 μg/mL each in MeOH) and 65 μL of MeOH and analyzed by gas chromatography/mass spectrometry (GC/MS). A 100-fold methanolic dilution of the extract was used for high-performance liquid chromatography (HPLC) analysis.

A methanolic solution containing THC and CBD 40 mg/mL each was prepared for the validation of the vaporizers with cannabinoid standards. The resinous THC was carefully heated at 60°C with a hot air blower and weighed directly into a 5-mL volumetric flask, combined with CBD and dissolved in MeOH-CHCl_3_ 9:1.

### Vaporizer devices and general experimental setup

Four electrically-driven and one gas-powered vaporizers were validated. The benchtop Volcano Medic^®^ and Plenty Vaporizer^®^ were obtained from Storz & Bickel (Tuttlingen, Germany). The pocket-size devices Arizer Solo^®^ was purchased from Arizer Tech (Waterloo, Canada), DaVinci Vaporizer^®^ from Organicix (Las Vegas, USA), and Vape-or-Smoke^™^ from Elemental Technologies (Seattle, USA). The vaporizers were operated according to the manufacturer’s instructions. The temperature was set at 210°C for all electrically-driven vaporizers. The temperature of Vape-or-Smoke^™^ could not be controlled or monitored. The aspiration of the vapor through the SPE column was performed at 420 mbar for 3 min, followed by 1 min at 100 mbar for the pocket-size devices. 420 mbar were applied for the complete evacuation of the balloon of Volcano Medic^®^.

A THC-type cannabis with 4.61% total THC (THC_tot_: THC + THC formed by thermal decarboxylation from THC acids during GC/MS analysis) and a CBD-type cannabis with 2.60% CBD (CBD_tot_: CBD + CBD formed by thermal decarboxylation from CBD acids during GC/MS analysis) and 0.53% THC_tot_ were used for the vaporization experiments. Experiments were performed for each device with 50 mg of plant material in triplicates for both cannabis varieties. The same number of experiments was conducted with 2 mg of THC and CBD standards (50 μL of THC and CBD, 40 mg/mL each in MeOH-CHCl_3_ 9:1). Four different fractions were collected for each experiment: (i) the vapor, (ii) all device parts of the vaporizers, which were in contact with the sample or the vapor, such as the sample chamber and the mouthpiece, (iii) the residue after vaporization, and (iv) the polypropylene tube connecting the mouthpiece, or the balloon in case of Volcano Medic^®^, to the vacuum pump. The vapor was trapped on a SPE column (15-mL Chromabond cartridge filled manually with 1 g of LiChroprep RP-18) using a Büchi B-172 vacuum pump, equipped with a Büchi B-168 vacuum/distillation controller (Büchi Labortechnik, Flawil, Switzerland). The SPE eluates were then quantitated by GC/MS for THC, CBD and CBN. To further elucidate the efficiency of the decarboxylation and vaporization process, the residues in the sample chambers and connecting parts were after rinsing with MeOH-CHCl_3_ 9:1 also analyzed by GC/MS and additionally HPLC for neutral and acidic cannabinoids, respectively.

### Vaporization procedures

After applying 50 mg of cannabis to the sample compartment of *Volcano Medic*^®^ as a thin layer covering the whole grid and connecting the balloon with valve to the sample chamber, vaporization was performed according to the manufacturer’s instructions. The device was preheated at 210°C. The balloon was connected to the mouthpiece after complete filling and linked with a polypropylene tube of 8 cm length to the SPE column tightly connected to the vacuum system. The vacuum controller of the pump was set to 420 mbar and the entire volume of the balloon aspirated through the SPE column. Then, the SPE column was removed and eluted with about 4 mL of MeOH-CHCl_3_ 9:1 by using an Adsorbex SPE unit (Merck, Darmstadt, Germany). The eluate was then evaporated to dryness at 40°C under a gentle stream of nitrogen (TurboVap^®^ LV evaporator, Zymark, Oftringen, Switzerland). The residue was reconstituted in 0.5 mL of MeOH-CHCl_3_ 9:1, vortexed and diluted 1:10 with MeOH. To 10 μL of this dilution, 25 μL of IS and 65 μL of MeOH were added. This sample was then analyzed by GC/MS to quantitate THC, CBD and CBN. Mouthpiece and the sample chamber were rinsed carefully with about 5 mL of MeOH-CHCl_3_ 9:1, and separately the tube connecting the mouthpiece to the SPE column. The samples were evaporated under a stream of nitrogen at 40°C and reconstituted in 0.5 mL of MeOH-CHCl_3_ 9:1 as described above. A 10-fold dilution was prepared from each sample by adding 25 μL of IS and 65 μL of MeOH to 10 μL of the reconstituted sample. The residual plant material from the sample chamber was extracted with 0.5 mL of MeOH-CHCl_3_ 9:1 as described above for the quantitation of cannabis test material. 25 μL of IS and 65 μL of MeOH were added to 10 μL of the filtrated extract and analyzed by GC/MS. The same extract was diluted 1:10 with MeOH and subjected to HPLC analysis. For the validation of the device with cannabinoid standards, 50 μL of a standard solution (THC and CBD, 40 mg/mL each in MeOH-CHCl_3_ 9:1) was carefully dropped onto the metal liquid pad, especially designed by the manufacturer to vaporize liquids. The 2 screens were removed from the sample chamber and the liquid pad was mounted. The sample chamber was connected to the preheated Volcano Medic^®^ with the temperature set at 100°C and the ventilator was turned on for 45 s to eliminate the solvents. Then, the sample chamber was removed from the device and connected to the balloon, while the temperature was increased to 210°C. After reaching 210°C the sample chamber together with the balloon were reconnected to the device, the ventilator was turned on immediately and the same procedure applied as described for cannabis. Then, the liquid pad was covered with MeOH-CHCl_3_ 9:1 and sonicated for 15 min to extract remaining cannabinoids. A gentle stream of nitrogen was applied to evaporate the solvent at 40°C, the sample reconstituted in 0.5 mL of MeOH-CHCl_3_ 9:1 and analyzed by GC/MS as well as HPLC after 10-fold dilution as described for the other fractions.

The *Plenty Vaporizer*^®^ was operated at the highest temperature setting (level 7) corresponding to 210°C. For the validation with cannabis, 50 mg of the plant material was applied onto the sample chamber and fixed with the liquid pad. For the validation with the cannabinoid standards, 50 μL of the standard solution was applied onto a filter paper (Schleicher & Schuell, Dassel, Germany) cut to the size of the sample chamber. The solvent was evaporated under a gentle stream of nitrogen at room temperature and the filter paper placed in the sample chamber. The mouthpiece fitted at the end of the cooling tube of the device was removed and the tube directly connected to the SPE column. The sample-loaded chamber was mounted on the vaporizer. As soon as the temperature was exceeding 205°C the vacuum pump was switched on and the aspiration performed for 3 min at a continuous flow with the vacuum controller set to 420 mbar, followed by 1 min at 100 mbar. The collection and sample preparation of the different fractions was done as described for Volcano Medic^®^. Especially the lamellae of the cooling tube had to be rinsed very carefully. The filter paper used for the vaporization of standards was sonicated for 15 min with a sufficient volume of MeOH-CHCl_3_ 9:1. The solvent was evaporated under a gentle stream of nitrogen at 40°C, the sample reconstituted in 0.5 mL of MeOH-CHCl_3_ 9:1 and analyzed by GC/MS and HPLC.

For the validation of the *Arizer Solo*^®^, the temperature was set to the highest level (7), which according to the manufacturer’s instructions corresponds to 210°C. The straight glass tube was connected with an 8-cm polypropylene tube to the SPE column. An adequate piece of glass wool was placed in the tube to cover the relatively large holes in the glass septum, separating the upper part of the tube from the sample chamber, in order to avoid the plant material being sucked by the vacuum. For the validation with the cannabinoid standards, 50 μL (2 mg) of the methanolic solution was carefully applied onto a cotton pellet (10 ± 2 mg). The solvent on the pellet was eliminated at room temperature under a gentle stream of nitrogen. The heater was switched on and the glass tube, pre-filled with the test materials, mounted on the device and the vacuum pump started immediately after reaching the temperature. The aspiration of the vapor through the SPE column was performed, as described for the Plenty Vaporizer^®^, for 3 min at 420 mbar followed by 1 min at 100 mbar. Sample fractions were obtained and processed as described in detail for Volcano Medic^®^. The cotton pellet used for the vaporization of standards was extracted with a sufficient volume of MeOH-CHCl_3_ 9:1 by sonication for 15 min, followed by evaporation of the solvent under nitrogen at 40°C, reconstitution of the residue in 0.5 mL of MeOH-CHCl_3_ 9:1 and analysis by GC/MS and HPLC.

The *DaVinci Vaporizer*^®^ was operated at 210°C. The mouthpiece was connected with an 8-cm polypropylene tube to the SPE column. The sample chamber was loaded with 50 mg of cannabis. For the validation with cannabinoid standards, 50 μL (2 mg) of the standard solution was transferred to the oil can supplied together with the device. The oil can was inserted into the sample chamber. To remove the solvent, the heater was started with the temperature set at 100°C and the sample chamber left open for 1 min after the temperature was reached. For the vaporization of cannabis or cannabinoids, the sample chamber loaded with the test materials was closed and the heater switched on. The vacuum pump was started immediately after reaching 210°C and the collection of the vapor was performed as described above for the Plenty Vaporizer^®^. The device was disassembled and sample fractions collected, processed and analyzed as described in detail for Volcano Medic^®^.

For the validation of the *Vape-or-Smoke*^™^ 50 mg of cannabis or 2 mg (50 μL) of cannabinoid standards were applied onto the sample chamber. The same procedure using cotton pellets was used for the vaporization of cannabinoid standards as described for the Arizer Solo^®^. The mouthpiece of the vaporizer was connected with a 8-cm polypropylene tube to the SPE column, and the SPE column linked to the vacuum pump. The vacuum pump was operated at a continuous flow with the vacuum controller set to 420 mbar for 3 min while the vaporizer’s butane gas flame was lightened intermediately with 6 cycles per min of 3 s duration each. At the end, the vacuum controller was set to 100 mbar and the vaporization continued for 1 more min with the same intermediate procedure. Sample fractions were collected, processed and analyzed as described in detail for Volcano Medic^®^. The cotton pellets used for the vaporization of cannabinoid standards were extracted as described for the Arizer Solo^®^.

### GC/MS assay

An HP 5890 II gas chromatograph equipped with an HP 6890 sampler and an HP 5972 mass selective detector (MSD) (Agilent Technologies, Palo Alto, CA, USA) was used. One-μL aliquots were injected splitless onto an Agilent DB-1MS capillary column (25 m x 0.25 mm i.d., 0.25-μm film) after 0.5 min column equilibration and subjected to the following temperature program: 100°C for 1 min, at 25°C/min to 175°C, at 5°C/min to 200°C and finally at 10°C/min to 300°C (total run time 18.0 min). The temperature of injector, transfer line and ion source were 250, 280 and 166°C, respectively. Helium was used as the carrier gas at a constant flow rate of 1.0 mL/min (67 kPa at 100°C). The MS system was operated in the standard electron impact (70 eV, 35 μA emission current) combined with the selected ion monitoring mode (SIM). The MSD was operated between 3.5 and 18.0 min, recording 3 groups of ions corresponding to CBD (3.5–14.9 min), THC (14.9–15.6 min) and CBN (15.6–18.0 min). The qualifier (dwell time 60 ms) and quantifier ions (target ion underlined, dwell time 100 ms) for CBD and CBD-D3 (IS) were *m/z* 246 and 231 and 249 and 234, respectively. For THC and THC-D3 (IS) the qualifier (dwell time 40 ms) and quantifier ions (dwell time 80 ms) were *m/z* 314, 299 and 231 and 317, 302 and 234, respectively. For CBN and CBN-D3 (IS) the qualifier (dwell time 40 ms) and quantifier ions (dwell time 80 ms) were m/z 310, 238 and 295 and 313, 241 and 298, respectively. The ions for CBD and THC were recorded with high resolution, whereas those of CBN were monitored with low resolution. Agilent enhanced ChemStation software G1701BA version B.00.00 was used for instrument operation, data registration and analysis. Quantitation was based on internal calibration and non-weighed linear regression analysis (least-squares model), calculating the peak area ratios of non-deuterated *vs*. deuterated analytes (IS). Calibrators and quality control samples containing THC, CBD and CBN were prepared from the commercial standard solutions *via* dilution with MeOH and the respective deuterated standards added as IS at 10 μg/mL. Three calibration sets were prepared and measured on 3 different days covering 1–250 μg/mL for CBD and CBN and 2–250 μg/mL for THC. Nine calibrator concentrations were used for CBD and CBN (1, 2, 5, 10, 20, 50, 100, 150 and 250 μg/mL) and 8 (2, 5, 10, 20, 50, 100, 150 and 250 μg/mL) for THC. A combined calibration curve was calculated for each compound from the mean peak area ratios of the 3 calibrators of each concentration. Four levels of control samples (3, 15, 40 and 130 μg/mL) were used to validate the assay and monitor the stability of the method during sample analysis. Method validation was performed by determining linearity range, accuracy, precision, and lower limit of quantitation (LLOQ; S/N = 10). Intraday (n = 5) and interday (n = 5) precision and accuracy data were obtained with control samples using 4 concentration levels (3, 15, 40 and 130 μg/mL). Peak assignment was based on retention times (CBD 14.5, THC 15.3, CBN 15.9 min) and mass spectra.

### HPLC assay

An Agilent 1100 series HPLC system (Agilent Technologies, Palo Alto, CA, USA) consisting of degasser, capillary pump, photodiode detector (PDA), column thermostat and temperature-controlled micro-automated liquid sampler was used for the quantification of THCA-A, CBDA, THC, CBD and CBN. Ten-μL aliquots were injected onto a 125 x 4 mm i.d. Spherisorb ODS I (3-μm particle size) column (Macherey-Nagel, Oensingen, Switzerland) equipped with a C8 Nucleosil precolumn (3 μm particle size; Macherey-Nagel). Separation was performed at 40°C and detection at 230 nm with the band width set to 4 nm. For peak assignment, PDA spectra were recorded from 190 to 400 nm. A binary solvent system consisting of MeOH (A) and 0.1% acetic acid (B) was used. Before each batch analysis, the column was equilibrated for 30 min with 50% (A). The flow rate was increased every 5 min with an increment of 0.15 mL/min from 0.1 to 0.7 mL/min, resulting in a pressure of 180 bar. The linear gradient was as follows: 50% (A) to 90% (A) at 20 min, kept at 90% (A) for 1.5 min and reduced to 50% (A) at 22 min. The HP ChemStation software for LC 3D Rev. A.09.03 was used for instrument operation, data recording and evaluation. Quantitation was based on external calibration using linear regression analysis (least-squares model). Combined calibrators and quality control samples containing all 5 analytes were prepared in MeOH by diluting the commercial standard solutions to the final concentrations. Three sets of calibrators were prepared and measured on 3 different days to calculate mean calibration curves for each compound from the mean peak areas on each concentration level. The calibration ranges were 1–250 (8 calibrators: 1, 2, 5, 10, 25, 50, 100, and 250 μg/mL), 0.4–250 (9 calibrators), 0.4–50 (7 calibrators), 1–500 (9 calibrators) and 0.4–250 μg/mL (9 calibrators) for CBD, CBDA, CBN, THC and THCA-A, respectively. To validate the assay and to monitor the stability of the system during sample analysis, 4 quality control samples (2.5, 20, 40, and 80 μg/mL) were used for CBD, CBDA and THCA-A, the 3 lower concentrations for CBN and an additional one (400 μg/mL) for THC. The same assay performance parameters as described for the GC/MS method were used to characterize the HPLC assay.

## Results and Discussion

### Assay validation

The GC/MS and HPLC methods for determination of cannabinoids were validated according to FDA validation guidelines [33]. The assays showed to be robust, selective, reproducible, accurate and sensitive. Intraday and interday precision and accuracy (bias) data are summarized in Supporting Information S1 and S2 Tables.

### Quantitation of test materials (cannabis and cannabinoid standards)

**Fig 1:**
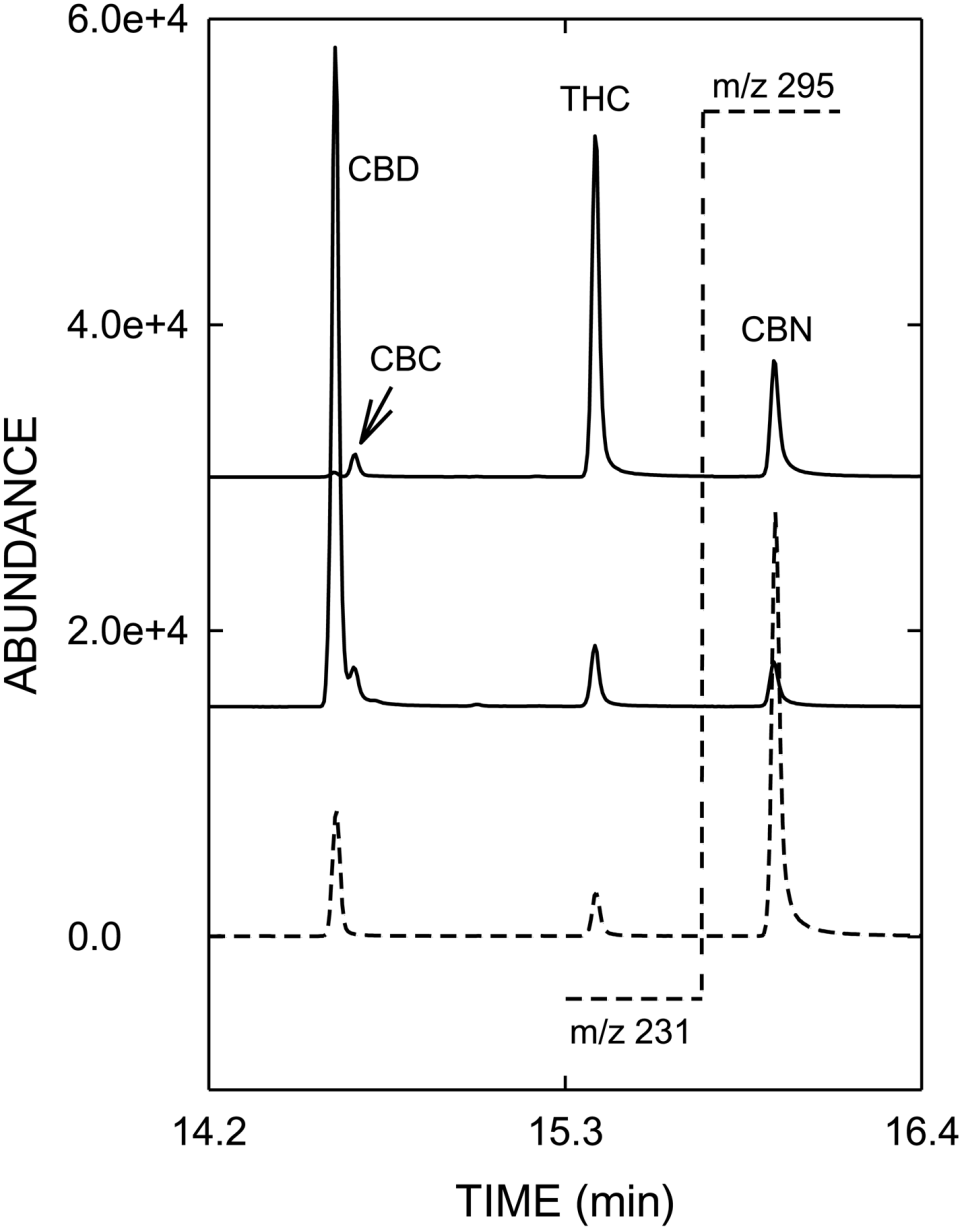
GC/MS-SIM profiles (from bottom to top) of an analytical standard mixture, CBD type cannabis and THC-type cannabis. The target-ion traces shown are m/z 231 for CBD and THC (left side of the dotted line at 15.6 min), m/z 295 for CBN (right side of the dotted line). Abbreviations: CBC = cannabichromene, CBD = cannabidiol, CBN = cannabinol, THC = delta-9-tetrahydrocannabinol.

**Fig 2:**
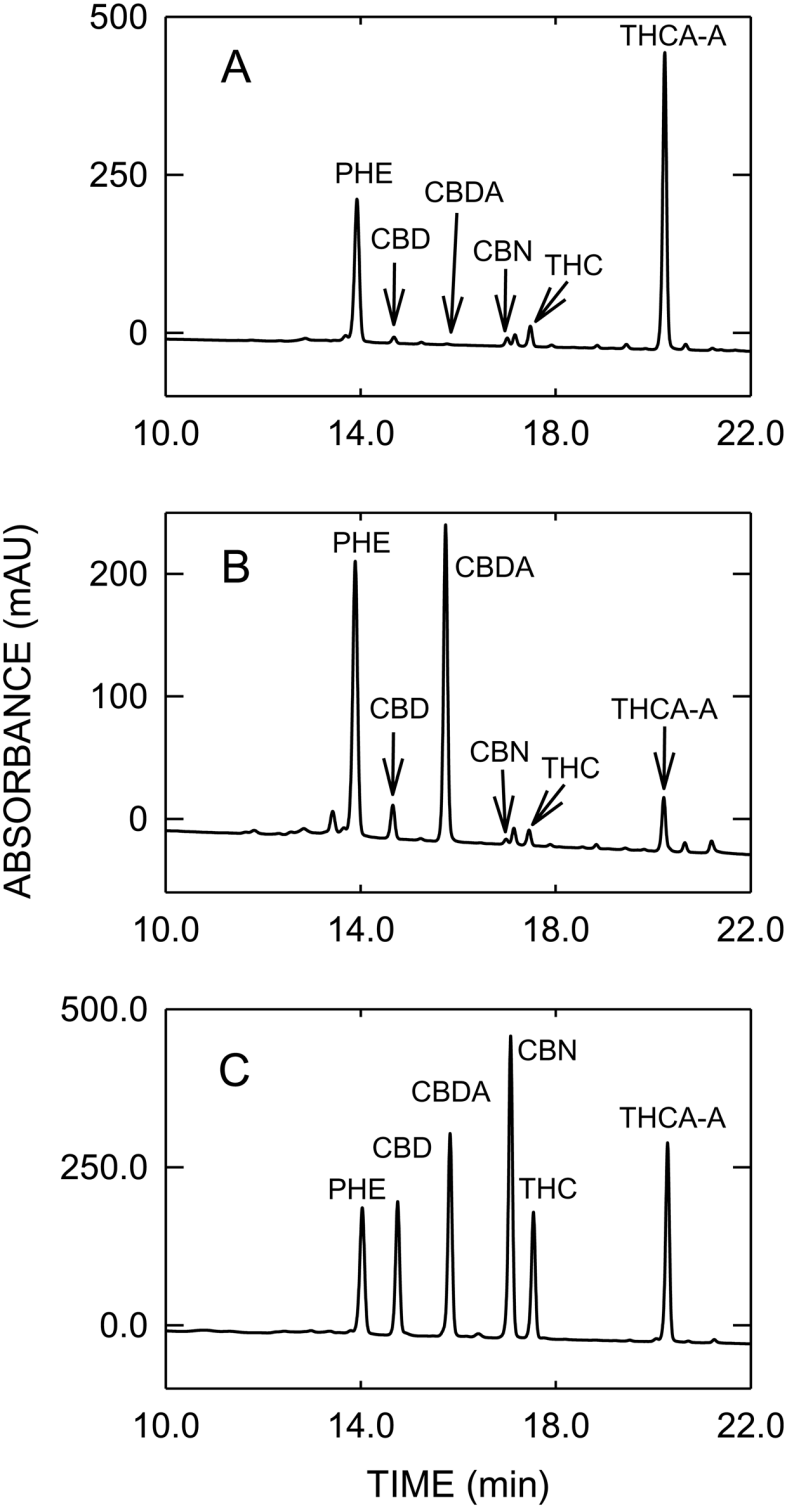
HPLC profiles of THC-type cannabis (A), CBD-type cannabis (B) and an analytical standard mixture (C). Abbreviations: PHE = phenanthrene, CBD = cannabidiol, CBDA = cannabidiol acid, CBN = cannabinol, THC = delta-9-tetrahydrocannabinol, THCA-A = delta-9-tetrahydrocannabinol acid A.

The contents of cannabinoids in THC- and CBD-type cannabis used for validation of vaporizers, determined by GC/MS and HPLC, are summarized in Supporting Information S3 Table, whereas typical GC/MS and HPLC profiles are shown in Figs 1 and 2, respectively. HPLC analysis revealed that 90.4% of THC_tot_ was present as THCA-A in the THC-type cannabis, whereas in the CBD-type cannabis CBDA was accounting for 85.8% of CBD_tot_. The concentrations of THC_tot_ and CBD_tot_ determined with HPLC were about 20% higher compared to those obtained with GC/MS, which can be explained by the incomplete thermal decarboxylation of acidic cannabinoids in the GC injector as described by Dussy and colleagues [34]. The content of CBN as a degradation product of THC was low in the THC-type and no CBN was detected in the CBD-type cannabis.

### *In vitro* validation of vaporizers

**Fig 3:**
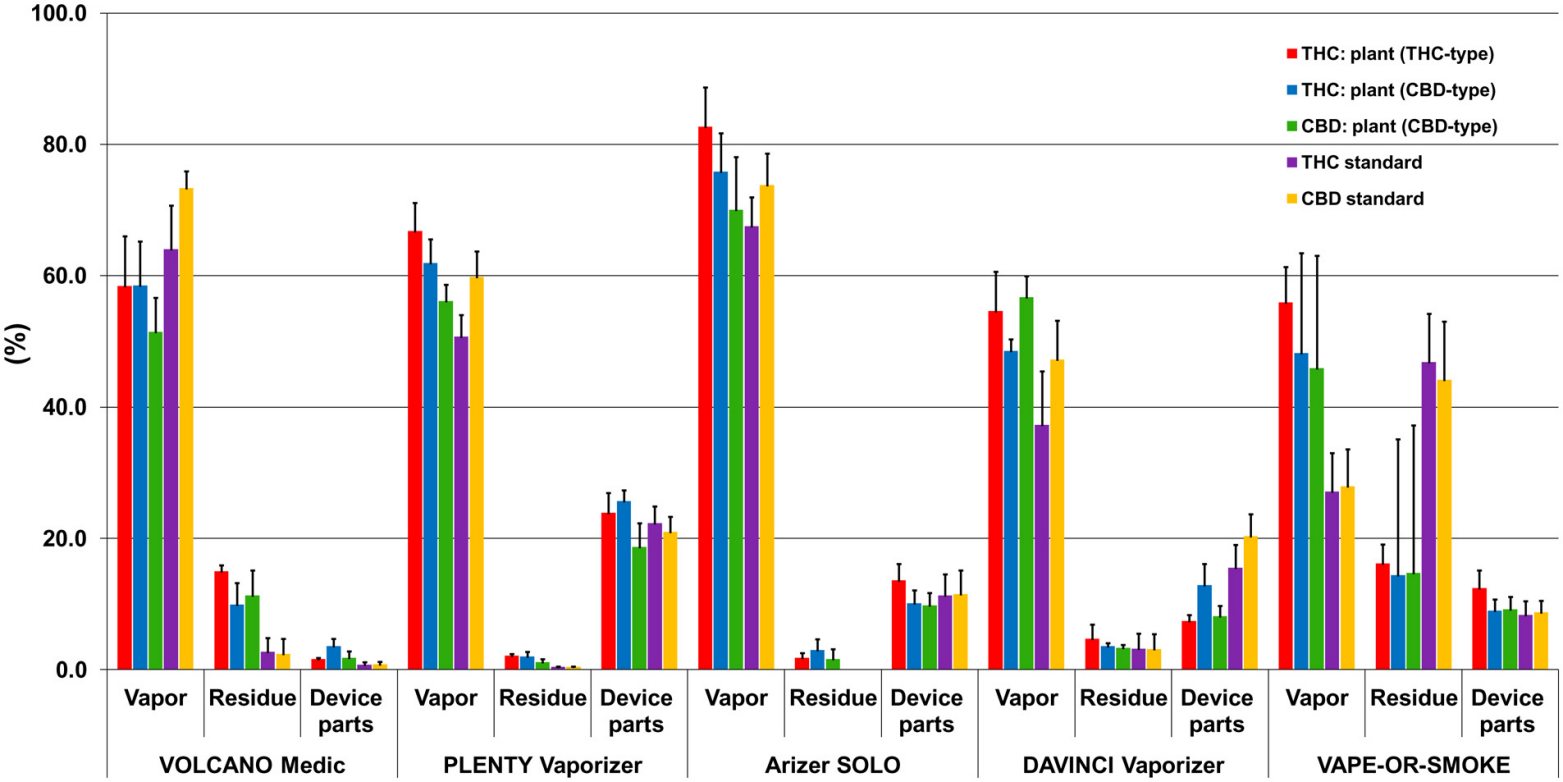
Percentage of total cannabinoids found in the vapor, residue and device parts of Volcano Medic^®^, Plenty Vaporizer^®^, Arizer Solo^®^, DaVinci Vaporizer^®^, and Vape-or-Smoke^™^. Depicted are the mean values + 1 SD. Bars, left to right: THC from THC-type cannabis; THC from CBD-type cannabis; CBD from CBD-type cannabis; THC standard; CBD standard.

**Fig 4:**
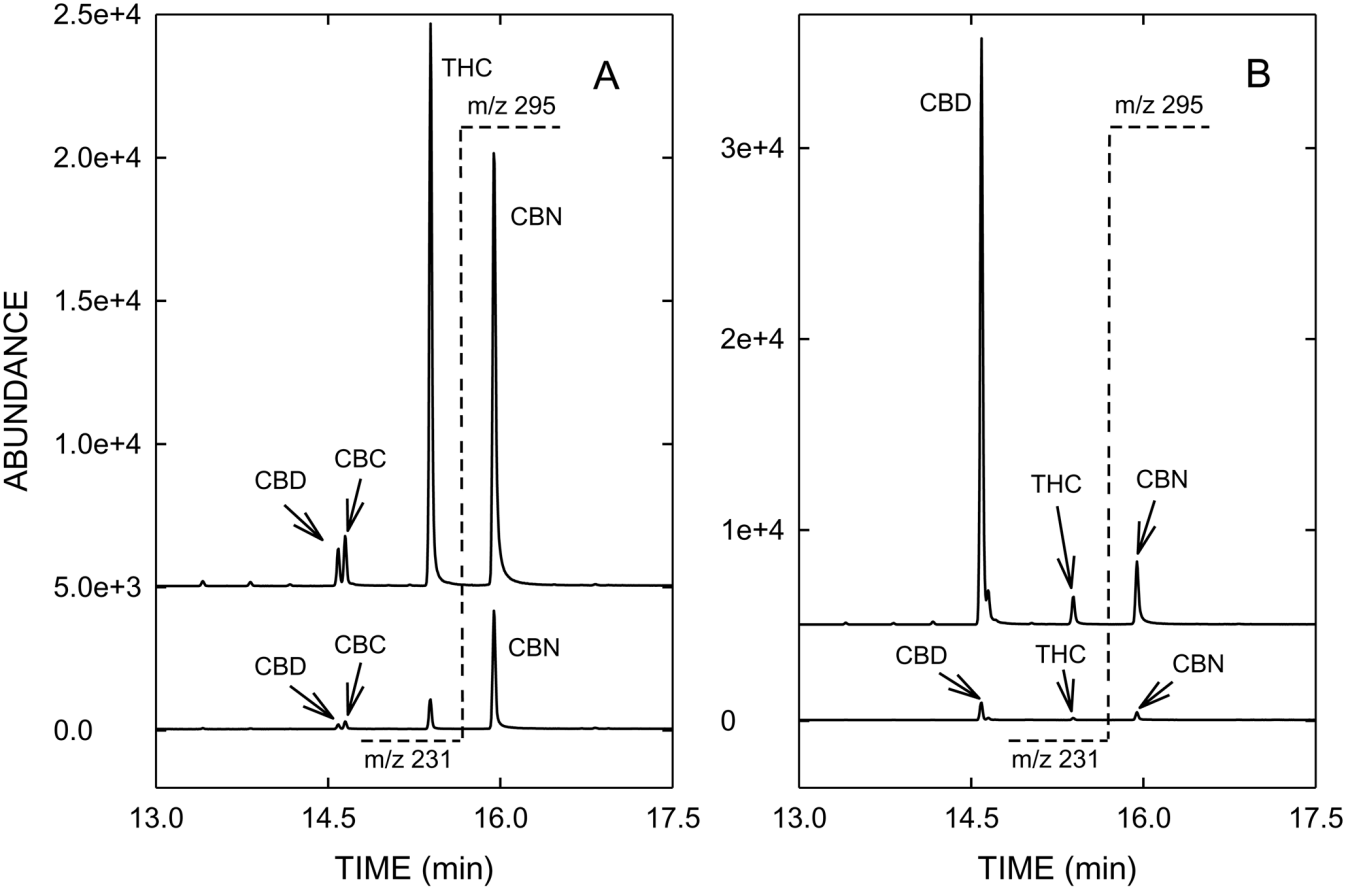
GC/MS-SIM profiles of the vapor (top graphs) and residue (bottom graphs) after vaporization of THC-type cannabis (A) and CBD-type cannabis (B) using the DaVinci Vaporizer^®^. The target ion m/z 231 of CBD and THC is plotted on the left side of the dotted line at 15.6 min, whereas m/z 295 as the target ion of CBN is depicted on the right side of the dotted line. Abbreviations as in Fig 1.

The recovery data, i.e. the cannabinoid concentrations measured in the sample fractions (vapor, residue, vaporizer parts) of the 5 vaporizers tested are summarized in Table 1 and Fig 3. The data of the connection tube are not included in Fig 3 as only traces (< 1%) or no cannabinoids were detected. Fig 4 depicts typical GC/MS profiles of vapor (top graphs) and residue (bottom graphs) obtained with THC-type (A) and CBD-type cannabis (B).

**Table 1:** Percentage of total cannabinoids found in the various fractions collected from 5 vaporizer devices.

Vaporizer Device	Fraction	Cannabis [Mean ± SD (RSD)^1^]			Standards [Mean ± SD (RSD)^1^]	
		THC-type	CBD-type			
		THC (%)	THC (%)	CBD (%)	THC (%)	CBD (%)
Volcano Medic^®^	Vapor	58.4 ± 7.6 (13.0)	58.5 ± 6.7 (11.4)	51.4 ± 5.2 (10.2)	64.0 ± 6.7 (10.5)	73.3 ± 2.6 (3.6)
	Residue	15.0 ± 0.9 (6.0)	9.9 ± 3.3 (33.3)	11.3 ± 3.8 (33.2)	2.7 ± 2.1 (76.2)	2.4 ± 2.3 (94.2)
	Device parts	1.6 ± 0.2 (15.1)	3.6 ± 1.1 (29.2)	1.8 ± 1.0 (56.9)	0.7 ± 0.4 (52.1)	0.8 ± 0.4 (59.0)
	Connection tube	< LLOQ	< LLOQ	< LLOQ	< LLOQ	< LLOQ
	*Sum*	*74*.*9 ± 6*.*9 (9*.*2)*	*72*.*0 ± 6*.*7 (9*.*3)*	*64*.*6 ± 2*.*9 (4*.*5)*	*67*.*4 ± 7*.*7 (11*.*4)*	*76*.*5 ± 3*.*2 (4*.*2)*
Plenty Vaporizer^®^	Vapor	66.8 ± 4.3 (6.5)	61.9 ± 3.6 (5.7)	56.1 ± 2.5 (4.5)	50.7 ± 3.3 (6.5)	59.8 ± 3.9 (6.4)
	Residue	2.1 ± 0.3 (12.7)	2.0 ± 0.7 (34.1)	1.1 ± 0.5 (43.0)	0.4 ± 0.1 (14.5)	0.4 ± 0.1 (21.0)
	Device parts	23.9 ±3.0 (12.6)	25.7 ± 1.6 (6.1)	18.7 ± 3.6 (19.4)	22.3 ± 2.6 (11.5)	21.0 ± 2.3 (11.1)
	Connection tube	< LLOQ	< LLOQ	< LLOQ	< LLOQ	< LLOQ
	*Sum*	*92*.*8 ± 4*.*0 (4*.*3)*	*89*.*6 ± 4*.*5 (5*.*0)*	*75*.*8 ± 3*.*8 (5*.*0)*	*73*.*0 ± 4*.*4 (6*.*0)*	*81*.*2 ± 3*.*3 (4*.*1)*
Arizer Solo^®^	Vapor	82.7 ± 6.0 (7.3)	75.8 ± 5.9 (7.8)	70.0 ± 8.1 (11.6)	67.5 ± 4.4 (6.5)	73.8 ± 4.8 (6.5)
	Residue	1.8 ± 0.7 (41.8)	3.0 ± 1.6 (53.2)	1.6 ± 1.5 (94.2)	< LLOQ	< LLOQ
	Device parts	13.6 ± 2.5 (18.5)	10.1 ± 2.0 (19.5)	9.8 ± 1.9 (19.2)	11.3 ± 3.2 (28.4)	11.5 ± 3.6 (31.5)
	Connection tube	< LLOQ	< LLOQ	< LLOQ	< LLOQ	< LLOQ
	*Sum*	*98*.*1 ± 7*.*1 (7*.*3)*	*88*.*9 ±5*.*0 (5*.*6)*	*81*.*3 ± 5*.*1 (6*.*3)*	*79*.*0 ± 2*.*6 (3*.*2)*	*85*.*4 ± 1*.*6 (1*.*9)*
DaVinci Vaporizer^®^	Vapor	54.6 ± 6.0 (11.0)	48.5 ± 1.8 (3.6)	56.7 ± 3.2 (5.7)	37.3 ± 8.1 (21.7)	47.2 ± 5.9 (12.5)
	Residue	4.7 ± 2.2 (47.2)	3.6 ± 0.4 (11.9)	3.3 ± 0.5 (14.5)	3.2 ± 2.3 (71.3)	3.1 ± 2.3 (72.3)
	Device parts	7.4 ± 0.9 (11.6)	12.9 ± 3.2 (24.5)	8.1 ± 1.6 (20.1)	15.5 ± 3.5 (22.8)	20.3 ± 3.4 (17.0)
	Connection tube	0.8 ± 0.2 (26.1)	< LLOQ	1.3 ± 0.3 (25.2)	0.6 ± 0.1 (16.4)	0.8 ± 0.1 (15.5)
	*Sum*	*67*.*5 ± 6*.*5 (9*.*6)*	*63*.*3 ± 4*.*5 (7*.*1)*	*68*.*6 ± 4*.*6 (6*.*7)*	*56*.*7 ± 11*.*5 (20*.*3)*	*71*.*5 ± 8*.*9 (12*.*4)*
Vape-or-Smoke^™^	Vapor	55.9 ± 5.4 (9.7)	48.2 ± 15.2 (31.5)	45.9 ± 17.1 (37.2)	27.1 ± 5.9 (21.7)	27.9 ± 5.7 (20.5)
	Residue	16.2 ± 2.9 (17.8)	14.4 ± 20.7 (143.4)	14.7 ± 22.5 (152.7)	46.8 ± 7.4 (15.7)	44.1 ± 8.9 (20.2)
	Device parts	12.4 ± 2.7 (21.7)	9.0 ± 1.7 (19.0)	9.2 ± 1.9 (20.7)	8.3 ± 2.1 (25.3)	8.7 ± 1.8 (21.0)
	Connection tube	0.8 ± 0.1 (8.5)	< LLOQ	0.5 ± 0.2 (45.4)	0.7 ± 0.1 (8.4)	0.7 ± 0.1 (15.9)
	*Sum*	*85*.*4 ± 0*.*3 (0*.*3)*	*71*.*6 ± 7*.*1 (9*.*9)*	*70*.*3 ± 6*.*5 (9*.*2)*	*82*.*8 ± 2*.*4 (2*.*9)*	*± 2*.*1 (2*.*6)*

^1^ Mean and SD in μg/mL, RSD in %.

Using cannabis (both types) and *Volcano Medic*^®^ more than 58% of the THC_tot_ was found in the vapor, whereas the recovery of CBD_tot_ was with 51.4% slightly lower. On the other hand, the recovery of THC and CBD was with 64.0 and 73.3%, respectively, higher compared to cannabis when vaporizing cannabinoid standards, whereas the amount of cannabinoids remaining in the residue was considerably lower. The reason may be that the cannabinoid standards were in the sample chamber directly applied onto the metal liquid pad designed for liquids, facilitating the vaporization of standards. RSD values of THC_tot_ and CBD_tot_ in vapor samples were 10 to 13% and 3.6 to 10%, respectively. When vaporizing cannabinoid standards, the recovery of CBD was higher than that of THC. THC and CBD left in the residue after vaporization was ≤ 15% and < 3% for cannabis and cannabinoid standards, respectively. The variability of the cannabinoids left in the residue was dependent on the percentage of total cannabinoids found in these samples. Consistently, RSD values were highest and up to 94.2% for the cannabinoid standards with the lowest content remaining in the residue. Only a minor percentage of ≤ 3.6% of total cannabinoids was deposited onto the device parts (i.e. sample chamber, connection valve and mouthpiece). The cannabinoids adsorbed on the balloon were not measured, which may be one explanation why the sum of all fractions was found to be between 64.6 and 76.5% with RSD being < 11.4%.

With 50.7 to 66.8% the recoveries in the vapor of *Plenty Vaporizer*^®^ were similar to those of Volcano Medic^®^. For this inhalation device, no differences were observed between cannabis and cannabinoid standards. The RSD values were significantly lower than those of Volcano Medic^®^, indicating a reliable and quantitative release of the cannabinoids from the sample matrix. With 1.1 to 2.1% (cannabis) and 0.4% (cannabinoids) remarkably less cannabinoids were left in the residue after vaporization with Plenty Vaporizer^®^ compared to Volcano Medic^®^. However, a considerable proportion of total cannabinoids, ranging from 18.7 to 25.7%, was found in the device parts. Some vapor condensation (RSD 6.1–19.4%) resulting in deposition and loss of cannabinoids occurs in the long metal cooling tube with lamellae designed to cool the vapor before inhalation. The sum of cannabinoids found in all fractions was with 89.6%– 92.8% highest for THC vaporized from cannabis.

The highest recoveries were obtained with *Arizer Solo*^®^, namely 67.5–82.7%. No differences were seen between THC and CBD. Except for CBD vaporized from cannabis (RSD = 11.6%) the variability was small with RSD values typically being < 8%. Only a minor portion of ≤ 3% of the cannabinoids remained in the residue after vaporization of cannabis and no cannabinoids were found in the piece of cotton used to vaporize the cannabinoid standards. Additional 9.8%– 13.6% of THC_tot_ and CBD_tot_ were adsorbed onto the glass tube and the sample chamber of the device, resulting in an overall recovery of 79.0–98.1%. The sum of the cannabinoids found in the various fractions was highest for THC vaporized from cannabis and reached 88.9% in CBD-type and 98.1% in THC-type cannabis. The relatively simple design of the device consisting of smooth surfaces and inert materials such as metal and glass may be responsible for the high yield.

The percentage of cannabinoids found in the vapor of the *DaVinci Vaporizer*^®^ varied between 48.5 to 56.7% and 37.3 to 47.2% for cannabis and cannabinoid standards, respectively. The oil can delivered with the DaVinci Vaporizer^®^ was used to vaporize the cannabinoid standards dissolved in MeOH. The insertion of the oil can into the sample chamber changes the design of the device as the oil can has to be heated passively by the sample chamber. This may be the reason for the lower recoveries obtained with the cannabinoid standards. Consistently, the variability was smaller with cannabis (RSD ≤ 11%) and bigger with the standards (< 22%). Less than 5% of the THC_tot_ and CBD_tot_ remained in the residue after vaporization of cannabis or standards. In case of cannabis, up to 12.9% of the THC and CBD was adsorbed onto the device parts. With 15.5–20.3% a higher proportion of THC and CBD was remaining on the device parts after vaporization of cannabinoid standards. This is a further indication that the insertion of the oil can into the sample chamber is causing a change in the heat distribution and/or temperature, therefore hindering the vaporization process. With ≤ 1.3%, only small amounts of cannabinoids were found in the connection tube. The overall recovery as the sum of all fractions was lower than that obtained with the other vaporizers, being between 56.7% and 71.5%. This may be the result of a limited sealing of the sample compartment.

The most important difference between the *Vape-or-Smoke*^™^ and all other vaporizers tested is that the Vape-or-Smoke^™^ is not electrically heated but with a butane gas flame. Consequently, the temperature cannot be adjusted or controlled. Therefore, it is difficult to maintain stable experimental conditions, which can be seen in the high variability of all measurements. 45.9% to 55.9% of the total cannabinoids present in cannabis were recovered from the vapor with RSD values between 9.7% and 37.2%. Only 27 to 28% of the cannabinoid standards were found in the vapor, which is most probably due to the experimental setup. As for the Arizer Solo^®^, the standards were vaporized from a piece of cotton introduced into the sample chamber. The lower part of the cotton turned brown during vaporization and isolated probably the upper part of the pellet, resulting in an incomplete vaporization of the cannabinoids. Consistently, a considerable amount of 44.1 to 46.8% of the cannabinoid standards remained in the residue. In case of cannabis, 14.4 to 16.2% of total cannabinoids were found in the residue. Again, the variability was with RSD values up to 152.7% extremely high, illustrating the difficulties in maintaining stable vaporization conditions. Overall, 70.3 to 85.4% of the total cannabinoids were found in all fractions together and no differences were found between the test materials.

**Fig 5:**
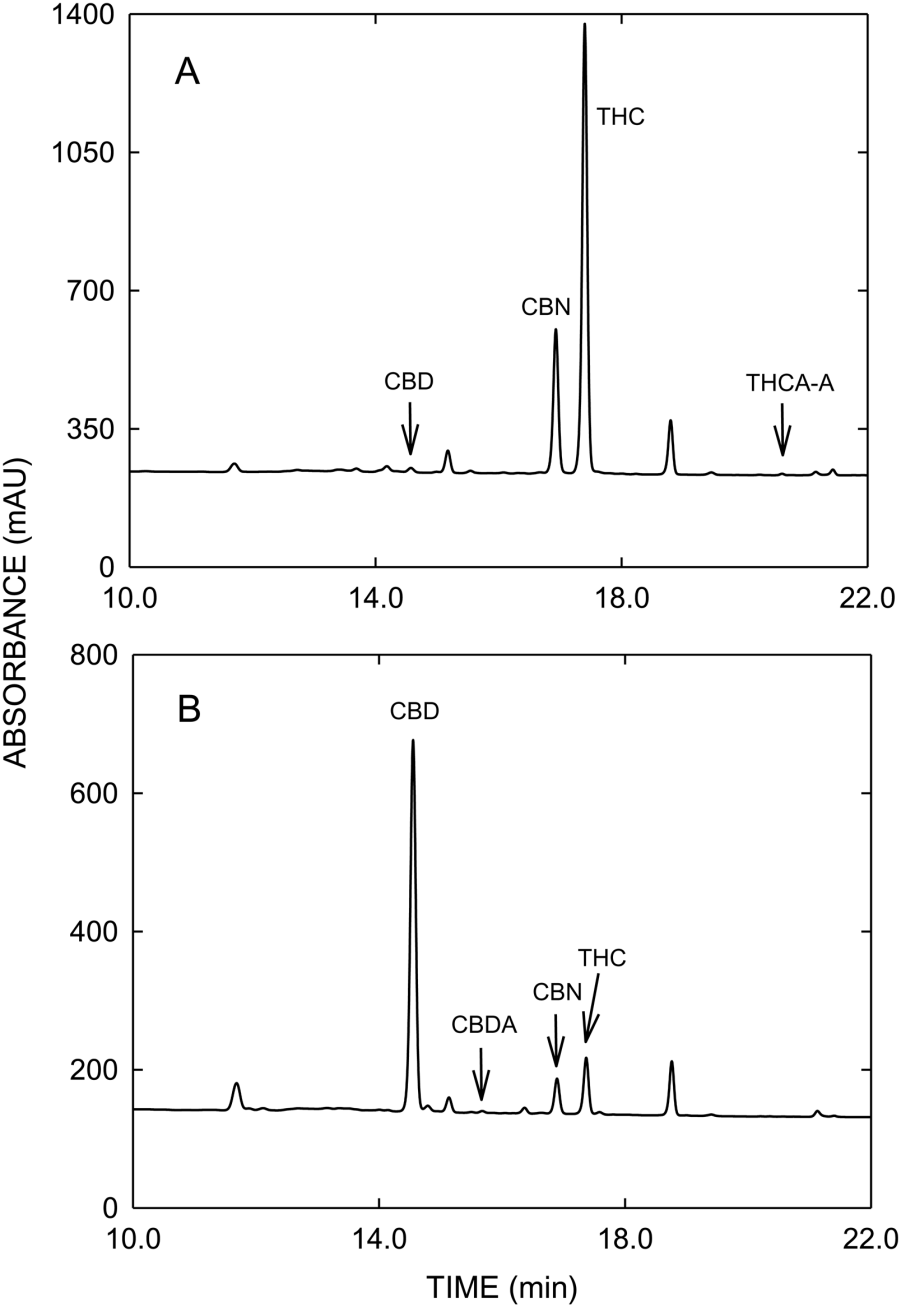
HPLC profiles of vapor samples collected from THC-type cannabis (A) and CBD-type cannabis (B) after vaporization with the DaVinci Vaporizer^®^. Abbreviations as in Fig 2.

As a consequence of the high vaporization temperature the concentration of CBN in vapor, formed by oxidation of THC, is slightly increased compared to cannabis (see Figs 1 and 4A). When vaporizing cannabis, an important issue is the efficient and quantitative decarboxylation of acidic cannabinoids, usually predominant in fresh cannabis, as neutral THC and CBD are considered to be pharmacologically active. In contrast to GC/MS, where derivatization (e.g. silylation) is required to prevent thermal decarboxylation of acidic cannabinoids, HPLC allows the direct analysis of acidic and neutral cannabinoids. The total decarboxylation rates achieved with the various vaporizers are summarized in Table 2. Excellent and reliable decarboxylation of THCA-A and CBDA was observed with all electrically-driven vaporizers, which allowed an exact control of the temperature. With ≥ 99.8% Plenty Vaporizer^®^ and DaVinci Vaporizer^®^ showed the highest decarboxylation efficiencies. With 97.3 to 98.7%, similar decarboxylation rates were found with Volcano Medic^®^ and Arizer Solo^®^, respectively. Only in case of CBD a slightly lower decarboxylation efficiency was found when vaporized with Volcano Medic^®^. On the other hand, Vape-or-Smoke^™^ operated with a butane gas flame showed lower decarboxylation of acidic cannabinoids with the respective rates being 87.7 to 93.3%. In addition, the variability of decarboxylation was generally found to be larger with this device with RSD values up to 12.7%, reflecting the difficulties in maintaining a constant temperature. Fig 5 with the corresponding HPLC profiles shows that in the THC-type cannabis THCA-A is almost completely converted to THC (A). The same holds true for CBD-type cannabis (B), i.e. the decarboxylation of CBDA to CBD.

**Table 2:** Decarboxylation efficiencies (%) of 5 vaporizer devices.

Vaporizer Device	THC-type Cannabis [Mean ± SD (RSD %)]	CBD-type Cannabis [Mean ± SD (RSD %)]	
	THC (%)	THC (%)	CBD (%)
Volcano Medic^®^	97.3 ± 1.1 (1.2)	97.3 ± 1.3 (1.3)	94.6 ± 2.2 (2.3)
Plenty Vaporizer^®^	99.8 ± 0.1 (0.1)	99.8 ± 0.1 (0.1)	99.8 ± 0.1 (0.1)
Arizer Solo^®^	98.6 ± 0.9 (0.9)	97.8 ± 1.7 (1.7)	98.7 ± 1.1 (1.1)
DaVinci Vaporizer^®^	99.9 ± 0.0 (0.0)	99.8 ± 0.1 (0.1)	99.8 ± 0.1 (0.1)
Vape-or-Smoke^™^	87.7 ± 2.3 (2.6)	93.3 ± 7.9 (8.5)	91.3 ± 11.6 (12.7)

## Conclusions

More and more interest has been directed towards the medical use of cannabis in the treatment or alleviation of a variety of symptoms in various diseases. As the oral administration of cannabinoids reveals poor and unreliable bioavailability and smoking of cannabis cannot be recommended for medical purposes, alternative efficient and less harmful application modes are needed. Vaporization of cannabis without the formation of potentially toxic pyrolysis products appears to be such an alternative. In this study, we performed an *in vitro* validation of 4 electrical and 1 gas-powered vaporizers regarding their ability to release THC and CBD with the vapor. The electrically-driven devices, enabling a precise control of the temperature, showed an almost complete decarboxylation of the acidic cannabinoids THCA-A and CBDA and good to excellent recoveries of the neutral cannabinoids THC and CBD in the vapor. Indications of combustion of cannabis, e.g. ash left in the sample compartment and visible smoke, was only found in the case of the gas-powered Vape-or-Smoke^™^. In addition, with this vaporizer unreliable decarboxylation and vaporization of cannabinoids was observed as a result of lacking temperature control. Therefore, gas-powered devices cannot be recommended for therapeutic purposes. Relative amounts of neutral cannabinoids released into the vapor varied considerably between the 4 electrically-driven devices. The largest difference was seen for THC vaporized from THC-type cannabis with 54.6% and 82.7% for the DaVinci Vaporizer^®^ and the Arizer Solo^®^, respectively. Here, the yield differed by 50% between the device with the lowest and that with the highest vaporizing efficiency. Lowest recoveries between 48.5–58.5% for cannabinoids from cannabis were obtained with the DaVinci Vaporizer^®^ and the Volcano Medic^®^, whereas the Plenty Vaporizer^®^ released 56.1–66.8% of total cannabinoids into the vapor. Best recoveries were obtained for the Arizer Solo^®^ with 70.0–82.7%. The better the recovery the less drug (cannabis or cannabinoids) is needed to deliver a defined therapeutic dose to a patient. This is an economic issue in terms of cost-effectiveness of the therapy with medicinal cannabis. Another aspect is the reliable and constant release of cannabinoids into the vapor to guarantee uniformity of the dosage, reflected by the RSD values obtained for the different devices. All electrically-driven vaporizers showed small variabilities with RSD values ≤ 13%. Excellent reproducibility was found for the Plenty Vaporizer^®^ (RSD ≤ 6.5%). It is also important to note that the design of the vaporizers had an impact on the yield of cannabinoids released with the vapor. Devices such as Volcano Medic^®^ and Plenty Vaporizer^®^ providing rather cold vapor for a mild, less airways-irritating inhalation revealed a smaller recovery of cannabinoids in the vapor compared to Arizer Solo^®^. However, this device, designed to release a maximum amount of cannabinoids into the vapor and lacking a cooling tube, produces a rather hot vapor, which may be less tolerated by patients.

In summary, the 4 electrically-driven and temperature-controlled vaporizers investigated in this study efficiently decarboxylate acidic cannabinoids and release reliably the corresponding neutral cannabinoids into the vapor. Therefore, they can be considered as a promising application mode for the safe and efficient administration of medicinal cannabis and cannabinoids. However, after the present *in vitro* validation clinical tests are required to confirm the efficiency of vaporizers as therapeutic application tools.

## Supporting Information

S1 Table GC/MS assay validation. (DOCX)

S2 Table HPLC assay validation. (DOCX)

S3 Table Quantitation of cannabinoid contents in THC- and CBD-type cannabis with HPLC and GC/MS. (DOCX)
