# Supplementary material for: Medicinal Cannabis: In Vitro Validation of Vaporizers for the Smoke-Free Inhalation of Cannabis
Source: PLoS One. 2016 Jan 19;11(1):e0147286. doi: 10.1371/journal.pone.0147286 (PMC4718604; doi:10.1371/journal.pone.0147286)
Supplement: S1 Table — (DOCX) [file pone.0147286.s001.docx]

**S1 Table. GC/MS assay validation**

|  | | **Intraday (n=5)** | | **Interday (n=5)** | |
| --- | --- | --- | --- | --- | --- |
| **Compound** | **Concentration** | **Precision** | **Accuracy** | **Precision** | **Accuracy** |
|  | (μg / mL) | Mean ± SD (RSD)^1^ | Bias (%)^2^ | Mean ± SD (RSD)^1^ | Bias (%)^2^ |
| CBD | 3.0 | 2.93 ± 0.02 (0.7) | - 2.2 | 2.91 ± 0.05 (1.7) | - 3.0 |
|  | 15.0 | 14.37 ± 0.08 (0.5) | - 4.2 | 14.37 ± 0.24 (1.7) | - 4.2 |
|  | 40.0 | 36.50 ± 0.45 (1.2) | - 8.7 | 39.43 ± 0.61 (1.6) | - 1.4 |
|  | 130.0 | 121.52 ±1.38 (1.1) | - 6.5 | 124.18 ± 2.77 (2.2) | - 4.5 |
| THC | 3.0 | 3.07 ± 0.07 (2.3) | + 2.3 | 3.19 ± 0.03 (0.9) | + 6.3 |
|  | 15.0 | 15.30 ± 0.15 (1.0) | + 2.0 | 14.56 ± 0.11 (0.8) | - 3.0 |
|  | 40.0 | 39.65 ± 0.36 (0.9) | - 0.9 | 39.72 ± 0.67 (1.7) | - 0.7 |
|  | 130.0 | 135.55 ± 1.51 (1.1) | + 4.3 | 130.91 ± 0.27 (0.2) | + 0.7 |
| CBN | 3.0 | 3.26 ± 0.19 (5.8) | + 8.7 | 3.03 ± 0.04 (1.5) | + 0.9 |
|  | 15.0 | 14.79 ± 0.30 (2.0) | - 1.4 | 14.89 ± 0.18 (1.2) | - 0.7 |
|  | 40.0 | 37.55 ± 0.56 (1.5) | - 6.1 | 40.80 ± 0.55 (1.3) | + 2.0 |
|  | 130.0 | 136.87 ± 2.60 (1.9) | + 5.3 | 134.38 ± 3.11 (2.3) | + 3.4 |

^1^ Mean and SD in μg/mL, RSD in %.

^2^ Deviation from the target value.
