# Supplementary material for: Medicinal Cannabis: In Vitro Validation of Vaporizers for the Smoke-Free Inhalation of Cannabis
Source: PLoS One. 2016 Jan 19;11(1):e0147286. doi: 10.1371/journal.pone.0147286 (PMC4718604; doi:10.1371/journal.pone.0147286)
Supplement: S2 Table — (DOCX) [file pone.0147286.s002.docx]

**S2 Table. HPLC assay validation**

|  | | **Intraday (n=5)** | | **Interday (n=5)** | |
| --- | --- | --- | --- | --- | --- |
| **Compound** | **Concentration** | **Precision** | **Accuracy** | **Precision** | **Accuracy** |
|  | (μg / mL) | Mean ± SD (RSD)^1^ | Bias (%)^2^ | Mean ± SD (RSD)^1^ | Bias (%)^2^ |
| CBD | 2.5 | 2.44 ± 0.04 (1.4) | - 2.3 | 2.45 ± 0.05 (2.0) | - 1.8 |
|  | 20.0 | 17.96 ± 0.15 (0.8) | - 10.2 | 18.35 ± 0.43 (2.4) | - 8.3 |
|  | 40.0 | 39.89 ± 0.20 (0.5) | - 0.3 | 39.45 ± 1.45 (3.7) | - 1.4 |
|  | 80.0 | 77.81 ± 0.43 (0.6) | -2.7 | 79.64 ± 2.69 (3.4) | - 0.5 |
| CBDA | 2.5 | 2.36 ± 0.01 (0.5) | - 5.6 | 2.35 ± 0.05 (1.9) | - 5.9 |
|  | 20.0 | 17.89 ± 0.16 (0.9) | - 10.6 | 17.91 ± 0.39 (2.2) | - 10.5 |
|  | 40.0 | 40.19 ± 0.19 (0.5) | 0.5 | 38.50 ± 1.36 (3.5) | - 3.8 |
|  | 80.0 | 81.91 ± 0.64 (0.8) | 2.4 | 78.88 ± 2.39 (3.0) | - 1.4 |
| CBN | 2.5 | 2.17 ± 0.02 (1.0) | - 13.0 | 2.22 ± 0.04 (1.9) | - 11.0 |
|  | 20.0 | 18.23 ± 0.17 (1.0) | - 8.9 | 18.56 ± 0.40 (2.2) | - 7.2 |
|  | 40.0 | 38.42 ± 0.25 (0.7) | - 3.9 | 37.67 ± 1.13 (3.0) | - 5.8 |
| THC | 2.5 | 2.47 ± 0.02 (0.6) | - 1.2 | 2.45 ± 0.04 (1.8) | - 2.0 |
|  | 20.0 | 18.03 ± 0.15 (0.8) | - 9.8 | 18.56 ± 0.65 (3.5) | - 7.2 |
|  | 40.0 | 39.00 ± 0.24 (0.6) | - 2.5 | 38.69 ± 1.55 (4.0) | - 3.3 |
|  | 80.0 | 75.32 ± 0.62 (0.8) | - 5.9 | 78.20 ± 2.78 (3.6) | - 2.3 |
|  | 400.0 | 436.96 ± 3.01 (0.7) | 9.2 | 419.64 ± 1.54 (0.4) | + 4.9 |
| THCA-A | 2.5 | 2.28 ± 0.07 (3.0) | - 9.0 | 2.41 ± 0.09 (3.6) | - 3.8 |
|  | 20.0 | 18.00 ± 0.18 (1.0) | - 10.0 | 18.09 ± 0.37 (2.1) | - 9.5 |
|  | 40.0 | 40.34 ± 0.30 (0.7) | 0.85 | 38.46 ± 1.25 (3.3) | - 3.9 |
|  | 80.0 | 83.23 ± 0.62 (0.7) | 4.0 | 79.33 ± 2.20 (2.9) | - 0.8 |

^1^ Mean and SD in μg/mL, RSD in %.

^2^ Deviation from the target value.
