# Supplementary material for: Medicinal Cannabis: In Vitro Validation of Vaporizers for the Smoke-Free Inhalation of Cannabis
Source: PLoS One. 2016 Jan 19;11(1):e0147286. doi: 10.1371/journal.pone.0147286 (PMC4718604; doi:10.1371/journal.pone.0147286)
Supplement: S3 Table — (DOCX) [file pone.0147286.s003.docx]

**S3 Table. Quantitation of cannabinoid contents in THC- and CBD-type cannabis with HPLC and GC/MS**

|  | | **Cannabinoid content (%)^1^**  **Mean ± SD (RSD)** | | | | |
| --- | --- | --- | --- | --- | --- | --- |
| **Cannabis** | **Method** | **CBD** | **CBDA** | **THC** | **THCA-A** | **CBN** |
| THC-type | HPLC | < LLOQ | < LLOQ | 0.53 ± 0.06 (11.5) | 4.97 ± 0.95 (19.2) | 0.09 ± 0.01 (12.3) |
|  | GC/MS | < LLOQ | n.a.^2^ | 4.61 ± 0.51 (11.0) | n.a.^2^ | 0.44 ± 0.04 (8.4) |
| CBD-type | HPLC | 0.46 ± 0.02 (3.4) | 2.77 ± 0.15 (5.6) | 0.20 ± 0.00 (1.7) | 0.48 ± 0.03 (5.9) | < LLOQ |
|  | GC/MS | 2.61 ± 0.05 (2.0) | n.a.^2^ | 0.53 ± 0.01 (2.4) | n.a.^2^ | < LLOQ |

^1^ % (w/w) of the dried plant material.

^2^ n.a.: not applicable, as in GC/MS acidic cannabinoids are decarboxylated.
